# A feasibility study for NOn-Traditional providers to support the management of Elderly People with Anxiety and Depression: The NOTEPAD study Protocol

**DOI:** 10.1186/s13063-018-2550-6

**Published:** 2018-03-07

**Authors:** Heather Burroughs, Bernadette Bartlam, Mo Ray, Tom Kingstone, Tom Shepherd, Reuben Ogollah, Janine Proctor, Waquas Waheed, Peter Bower, Peter Bullock, Karina Lovell, Simon Gilbody, Della Bailey, Stephanie Butler-Whalley, Carolyn Chew-Graham

**Affiliations:** 1 Research Institute for Primary Care and Health Sciences, Keele University, Staffordshire, ST5 5BG UK; 2 Department School of Health and Social Care, Lincoln University, Lincoln, UK; 3 South Staffordshire and Shropshire NHS Healthcare Foundation Trust, St Georges Hospital, Stafford, ST16 3SR UK; 4 National Institute of Health Research School for Primary Care Research, Centre for Primary Care, Institute of Population Health, University of Manchester, Manchester, UK; 5 Division of Nursing, Midwifery and Social Work, School of Health Sciences, Faculty of Biology, Medicine and Health, University of Manchester, Manchester Academic Health Science Centre, M13 9PL, Manchester, UK; 6 Department of Health Sciences, University of York, Heslington, York, YO10 5DD UK

**Keywords:** Depression, Anxiety, Older people, Psychosocial intervention, Community, Third sector

## Abstract

**Background:**

Anxiety and depression are common among older people, with up to 20% reporting such symptoms, and the prevalence increases with co-morbid chronic physical health problems. Access to treatment for anxiety and depression in this population is poor due to a combination of factors at the level of patient, practitioner and healthcare system.

There is evidence to suggest that older people with anxiety and/or depression may benefit both from one-to-one interventions and group social or educational activities, which reduce loneliness, are participatory and offer some activity. Non-traditional providers (support workers) working within third-sector (voluntary) organisations are a valuable source of expertise within the community but are under-utilised by primary care practitioners. Such a resource could increase access to care, and be less stigmatising and more acceptable for older people.

**Methods:**

The study is in three phases and this paper describes the protocol for phase III, which will evaluate the feasibility of recruiting general practices and patients into the study, and determine whether support workers can deliver the intervention to older people with sufficient fidelity and whether this approach is acceptable to patients, general practitioners and the third-sector providers.

Phase III of the NOTEPAD study is a randomised controlled trial (RCT) that is individually randomised. It recruited participants from approximately six general practices in the UK. In total, 100 participants aged 65 years and over who score 10 or more on PHQ9 or GAD7 for anxiety or depression will be recruited and randomised to the intervention or usual general practice care. A mixed methods approach will be used and follow-up will be conducted 12 weeks post-randomisation.

**Discussion:**

This study will inform the design and methods of a future full-scale RCT.

**Trial registration:**

ISRCTN, ID: ISRCTN16318986. Registered 10 November 2016. The ISRCTN registration is in line with the World Health Organization Trial Registration Data Set. The present paper represents the original version of the protocol. Any changes to the protocol will be communicated to ISRCTN.

**Electronic supplementary material:**

The online version of this article (10.1186/s13063-018-2550-6) contains supplementary material, which is available to authorized users.

## Background

Anxiety and depression are prevalent among older people, with up to 20% reporting symptoms of depression [1, 2]. Demographic changes mean that even if prevalence rates were to remain stable, the growing numbers of older people will translate into large increases in the demand for treatment for these disorders in this population [3]. This will place an increasing burden on health and social care.

Untreated anxiety and depression lead to increased use of health and social care services, and raised mortality [4]. Depression and anxiety are more prevalent in people with long-term physical conditions. The prevalence of depression in people with diabetes may be as high as 30% [5], and the prevalence of anxiety in people with chronic obstructive pulmonary disease is up to 25% [6]. Depression is more than seven times more common in those with two or more chronic physical conditions [5]. Thus, mental and physical health problems tend to become entwined and manifest in complex co-morbidity [7]. As co-morbidities are common in later life (36% of people aged 65–74 and 47% of those aged 75 and over have a limiting chronic illness), they constitute a serious risk factor for developing depression and/or anxiety. Treatment of depression has the potential to improve outcomes for diabetes [7] and to improve mortality from all causes in older adults [8].

Depression and anxiety in older people are poorly detected and managed in primary care [9]. This is particularly the case in people with chronic physical ill health problems [10]. One impediment to detection is that older people may not present to their general practitioner (GP) with depression because of the stigma they perceive about mental health problems [11, 12]. In addition, older people express a preference for talking treatments rather than antidepressants [13].

A one-to-one intervention for older people with depression may be insufficient. According to a systematic review of interventions for isolated and depressed older people [14], nine of the ten effective interventions included were group activities with an educational or support input, whereas six of the eight ineffective interventions provided one-to-one social support, advice and information, or a health-needs assessment. There is a body of evidence demonstrating the beneficial effects of social connectedness on psychological and physical well-being [15–17]. Indeed, a meta-analysis [15] showed that stronger social relationships were associated with longer life expectancy and the magnitude of the effect was comparable to ceasing smoking, and it exceeded that for obesity, high blood pressure and physical inactivity (the random effects weighted average effect size had an odds ratio of 1.50 with a 95% confidence interval of 1.42 to 1.59), indicating a 50% increased likelihood of survival for participants with stronger social relationships. Forsman’s systematic review [18] suggests that meaningful social activities, tailored to the older individual’s abilities and preferences, should be considered in aiming to improve mental health and well-being among older people. A further argument for increasing social participation is that loneliness and depression are strongly associated in older people [19, 20] and loneliness is an independent risk factor for depression [21]. Evidence from the United States [22] suggests that for lonely older people, there is a potential benefit from group social or educational activities. Thus, it is reasonable to postulate that group activity might be a useful adjunct to treatment for mild to moderate depression. Wicke et al. [23] demonstrate that social support influences health-related quality of life and that this association is strongly mediated by depressive mood, and they suggest that interventions for patients with depression and multi-morbidities should address social dimensions.

Further evidence from a systematic review of social interventions targeting loneliness in older people [24] suggests that the most successful interventions for loneliness, measured by improvement in the domains of physical, mental and social health, tend to be group based, participatory and offering some activity [25–27]. Such community-based interventions have been shown to have additional benefits in terms of social inclusion and social cohesion [28–30]. Despite the growing call for a diverse range of support for older people, there remains a paucity of evidence on what works best in reaching those who are most vulnerable [31] and it is clear that better designed studies, and in particular randomised controlled trials (RCTs), are needed to improve the evidence base [24].

Behavioural activation (BA) (a short-term intervention based on cognitive behavioural therapy) is a talking treatment known to be effective in the management of depression [32, 33]. BA focuses on activity scheduling to encourage participants to engage in activities that they may have previously enjoyed but are currently avoiding. In addition, it helps participants to develop new activities that can accommodate changes in life circumstances such as losses (e.g. spousal bereavement). Also, BA encourages participants to be aware of the presence and effects of cognitive processes (e.g. rumination) that may serve to reinforce avoidance and/or lack of engagement. Participants are, thus, supported to refocus on their goals and valued directions in life. The main advantage of BA-oriented interventions over traditional cognitive behavioural therapy for depression is that it may be easier to train non-clinical staff in it [34]. In addition, behavioural therapies have been shown to be effective in older people [35, 36]. BA forms the cornerstone of a number of recent trials where the intervention is delivered by psychological well-being practitioners. Richards et al. [37] found that the intervention for adults of all ages, within a collaborative care model, was effective.

### Rationale

The study is in three phases and phases I and II have been completed. In phase I, we synthesised existing guidelines, results from an updated systematic review and results from new empirical qualitative research findings. This synthesis enabled us to refine our BA psychosocial intervention. It is designed for older people with anxiety and/or depression and will be deliverable by third-sector support workers. In phase II, we assessed the feasibility of recruiting and training third-sector practitioners (support workers) to deliver the intervention.

## Methods

### Aims

The aims of phase III of the study are (1) to determine if it is feasible to recruit and randomise patients, (2) to pilot procedures and (3) to conduct a process evaluation to provide essential information and data to inform a proposal for a full randomised trial.

The objectives are

1. To assess feasibility in terms of recruitment of GPs and participants, retention and delivery of the intervention
2. To assess the acceptability of the intervention to participants, support workers and GPs
3. To assess questionnaire completion rates (both arms) and non-compliance with the intervention

### Design and setting

The SPIRIT (Standard Protocol Items for Randomised Trials) recommendations were followed in preparing this protocol (see Additional file 1). This is an individually randomised feasibility study identifying approximately 100 eligible participants from at least six general practices in North Staffordshire, UK.

General practices are eligible to take part if they use the clinical operating system EMIS Web. This is a clinical electronic computer system for delivering healthcare, which allows healthcare professionals to record, share and use vital patient information. The general practices will be recruited through the Clinical Research Network: West Midlands of the National Institute for Health Research (NIHR). Practice recruitment will be staggered, beginning with searches and a mail to potential participants from two practices, before we gradually increase the number of practices as necessary. The participation of general practices will be formalised through written service level agreements.

### Sample size

**Fig. 1:**
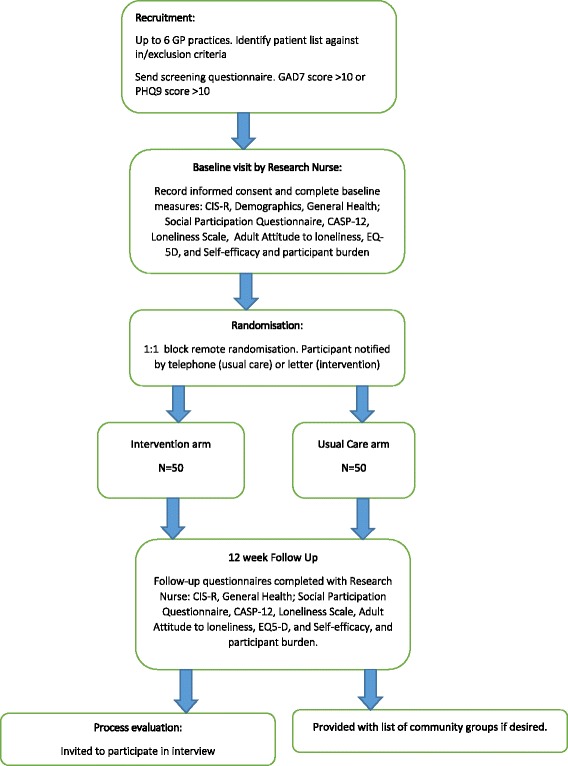
NOTEPAD CONSORT flow chart

As this is not a hypothesis testing trial, a formal power calculation is not required. Nonetheless, as this is a feasibility study, each arm will consist of 30–40 participants to estimate reliably process outcomes relating to recruitment, retention and attrition rates to inform a fully powered RCT [38]. We anticipate that the total combined loss to follow-up will not exceed 20% at 4 months and therefore, we aim to recruit 50 participants to each treatment arm (Fig. 1).

We aim to recruit up to six practices to enable us to recruit the required number of participants. Approximately 1000 people over 65 are expected to be registered in an average-sized general practice of 5000 patients. On the assumption that 50% will respond to the postal screening questionnaire, of which we expect 20% will have anxiety and/or depression, we anticipate a potential sample size of 100 per practice. Of these, 30% might be expected to give consent to take part, resulting in an anticipated recruitment of approximately 30 participants per practice over a 9-month period. Confirming these screening and enrolment rates is part of the reason for performing the study.

### Participants

Practice lists from the general practices will be searched by research facilitators in the clinical research network for people aged over 65 years. GPs will screen the resulting lists and be asked to remove people who, as far as they are aware, meet our exclusion criteria.

Our exclusion criteria are:

- People who are actively suicidal or harming themselves
- People under the care of secondary or specialist mental health services
- People currently misusing alcohol or other substances
- People in the palliative phase of an illness
- People lacking the capacity to consent
- People unable to understand or read English
- People living in a care home

**Fig. 2:**
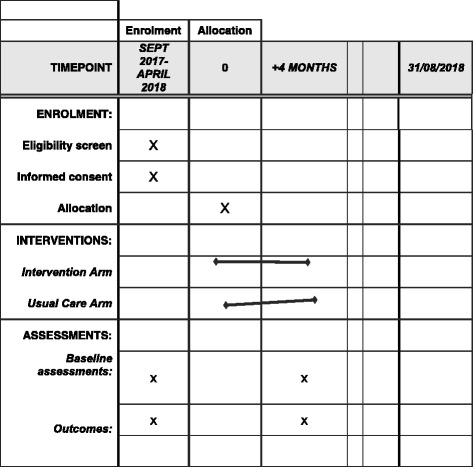
NOTEPAD: schedule of enrolment, interventions and assessments

The resulting potential participants will be sent an invitation pack consisting of an invitation letter, a participant information sheet, a postal screening questionnaire with a consent to contact form and a stamped addressed envelope (Fig. 2).

The postal screening questionnaire consists of the Patient Health Questionnaire (PHQ9) [39] and the Generalised Anxiety Disorder (GAD7) scale [40] and a section seeking consent for further contact. Individuals returning a completed questionnaire with a score of 10 or higher on either the PHQ9 or the GAD7 and who consent to further contact will form the sample for invitation into the feasibility study. A research nurse will contact these individuals and re-complete the PHQ9 and GAD7 over the phone to ensure their depression and anxiety symptoms are not transient. For those who score 10 or higher on either the PHQ9 and/or the GAD7, the research nurse will arrange a home visit for a baseline assessment. Those who score 9 or lower on both PHQ9 and GAD7 will be informed by the nurse at the time, over the telephone, that they are not eligible for the study and if they wish, they will be sent a copy of a resource directory of local social activity groups.

If no response to the postal screening questionnaire is received after 14 days, a reminder postcard will be sent to participants.

### Recruitment to the NOTEPAD trial

At the baseline home visit, the research nurse and potential participant will discuss the study further. Prior to the initial mail, the patients were screened against the exclusion criteria by their GP, but the research nurse will check the inclusion and exclusion criteria again and will also administer an assessment of cognitive capacity using a cognitive capacity proforma. If the participant has impaired cognitive capacity, they will not be recruited to the study.

From those eligible, the research nurse will explain what participating in the study will involve, i.e. the completion of baseline and follow-up questionnaires, and if randomised to the intervention arm, participation in up to six consultations with an Age UK support worker, a qualitative interview (sub-sample only) and audio recording of up to two consultations with the Age UK support worker.

### Randomisation

Once written informed consent and the baseline measures have been collected, participants will be randomised by the Keele Clinical Trials Unit (CTU) study administrator to either the intervention arm or the usual GP care arm, using third-party computerised randomisation supported by CTU. After the baseline visit, the research nurse will contact the study administrator, who will randomise the participant using a study-specific database. A researcher will inform those allocated to the usual care group of the outcome by telephone. If a participant is randomised to the intervention arm, they will receive a letter informing them that an Age UK support worker will make contact with them in the next week. The participant’s contact details will be sent to the Age UK support worker by safe haven fax.

Randomisation will be in a 1:1 ratio, using randomly permuted blocks of sizes 4, 6 and 8, to ensure a balanced allocation to each arm of the trial. The study statistician and the study team members involved in the collection of follow-up data will be blinded. Each participant’s GP will be sent a letter informing them of their patient’s participation and randomisation allocation.

### Allocation concealment, blinding and selection bias

A clinical research network nurse blind to subsequent treatment allocation will obtain informed consent. Selection bias at recruitment will be avoided by separating the processes of determining patient eligibility and intervention allocation. The trial database will be password protected to ensure that the trial statistician and study personnel involved in the questionnaire data collection remain blind to treatment allocation. Data entry, coding, security, storage and management will follow the standard operating procedures at Keele CTU.

### The intervention

Participants in the intervention arm will be offered an individual appointment with the Age UK support worker at a local third-sector service or in the participant’s home (depending on preference). It is likely that the intervention will be more acceptable if delivered in the participant’s own home [41]. There will be 4–6 contacts between the participant and the support worker, utilising a combination of face-to-face and telephone contact.

The components of the intervention include person-centred assessment and engagement, problem definition, risk assessment, mood monitoring, BA (a structured programme for reducing the frequency of negatively reinforced avoidant behaviours in parallel with increasing the frequency of positively reinforcing behaviours to improve functioning and improve mood) and signposting to local agencies and activities. If during treatment it becomes apparent that symptoms are not improving, the case manager and participant will collaboratively discuss options for further treatment. This may require referral back to the GP for possible medication review or a prescription or stepping up into the local Improving Access to Psychological Therapies service.

The intervention is intended to be tailored to patient preference so there is some flexibility regarding the precise number of sessions, interval, mode of delivery and format. Participant preference for the type of session will be explored in the qualitative process outcome interviews. The content of the intervention will be monitored for fidelity by audio recording the first two sessions a support worker has with each participant. These audio recordings will be transcribed and checked against a fidelity checklist as part of the process evaluation. Intervention group participants will also receive treatment as usual from their primary care team.

The participants and support workers will be asked to record basic information about participation activities. Details or referrals, signposting or accompanying participants to third-sector services will be recorded as part of the support worker role. The support worker will complete a brief summary of the content of each participant contact. We will ask the participants to record a one-line summary of their sessions with the support worker and activities that they attended (or been signposted to, but not attended). Our Patient and Public Involvement and Engagement (PPIE) group have helped identify how best to collect such data and the result has been the production of an A5 Filofax-style patient resource, which includes depression and anxiety information with example stories; a section to keep notes on the weekly sessions; a local resource directory, which is to be populated by the support worker with relevant information about activities in the local area; tips on staying well and relaxation; and a useful contacts section.

### Usual care

Participants randomised to the usual care arm will receive whatever care is judged to be indicated by the primary care practitioners in contact with them. We will use the feasibility study to describe care for participants in the usual care arm of the study. Data on self-reported health and social care utilisation will be collected at the 4-month follow-up. No constraints will be placed on what constitutes usual care. At the follow-up, participants in the usual care arm will be offered a list of local community groups, which they may choose to access.

### Development of the support worker training

The support worker training programme was delivered over 3 days by members of the research team and this included: the aims of the study, an overview of anxiety and depression in older people, principles of BA and how to deliver the intervention. Role play with simulated patients enabled the support workers to practise their skills. The training programme was augmented with a support worker handbook containing the training materials and supplementary background information.

Six support workers plus one reserve were trained to deliver the intervention and the training took place in a group setting over 3 days.

### Supervision of the support workers

The support workers will be supervised to ensure safe practice and adequate management of risk. In addition, they will meet informally as a group at least every 2 months to facilitate peer support.

### Outcomes

To achieve the aims and objectives set out for this study, we have taken a mixed methods approach, which includes validated measures at baseline and follow-up; a process evaluation consisting of semi-structured interviews with study participants, GPs and support workers; and audio recordings of the support worker consultations. These methods of data collection will help inform the design and methods of a future full-scale RCT and we can examine the feasibility, acceptability and fidelity of the support worker intervention.

Feasibility, acceptability and fidelity will be assessed using the measures below:

1. Engagement of general practices will be measured by recording the number of general practices that agree to participate of those approached.
2. Recruitment, training and retention of support workers will be measured by monitoring how many support workers undergo full training and are retained to the end of the study.
3. Response rates to the screening questionnaire will be measured by recording the number of target participants that respond to screening as a percentage of the number mailed and invited to participate in the study.
4. Participant recruitment rate will be measured by the number of eligible participants who consent to participate in the study as a percentage of all eligible participants.
5. Response rates to the follow-up questionnaire will be measured recording the number of participants who consent to participate that remain in the study until the end of follow-up at 4 months
6. Adherence to intervention will be measured by reviewing support worker notes at 4 months, recording of sessions and qualitative interviews with support workers and a sample of participants

The Computerised Clinical Interview Schedule Revised (CIS-R) [42] total score, administered via laptop, will be our primary clinical outcome at baseline and 4 months, but its performance will be examined in the pilot study in terms of its precision (based on the 95% confidence interval), completion rate at the item and scale level, and any evidence of floor or ceiling effects.

Secondary outcome measures include:

- General health questions
- Self-efficacy [43] (for those who disclose a long-term condition)
- Health-related quality of life (EQ-5D-5 L) [44]
- Quality of life (Control, Autonomy, Self-realisation and Pleasure or CASP-12) [45]
  Loneliness [46]
- Adult attitude to loneliness^1^ [47, 48]
- Social participation questionnaire [49]
- Participant burden: The level of burden will be monitored by free text, which will be analysed as part of the process evaluation and the optimum method of administration determined to help inform the methods of the full trial.

### Quantitative data

The analysis will follow a detailed statistical analysis plan formally agreed with the study steering committee prior to data analysis. The analysis will focus on: (i) describing the key process measures to decide if a main trial is feasible, (ii) a baseline description of the study sample, (iii) exploratory analysis of clinical outcomes, (iv) reports of adverse events in any of the treatment arms, (v) descriptive summaries of the contacts made with the support worker (in relation to adherence with randomised intervention) and satisfaction with care and (vi) extent of missing data and data accuracy. There will be no emphasis on hypothesis testing, which is reserved for the future main trial. Feasibility outcomes will be estimated using descriptive statistics (with 95% confidence intervals). The assessment of key process measures will include determining the engagement of general practices; recruitment, training and retention of support workers; response rate to the screening questionnaire; and participant recruitment uptake and response to follow-up. Key baseline characteristics (age, gender and index of multiple deprivation) will be compared between trial participants and the ineligible and non-consenting patients, to ascertain the adequacy of inclusion and exclusion criteria and likely generalisability of the trial to the required targeted population. Similarly, at the 4-month follow-up, we will compare the key patient characteristics between those followed up and those lost to follow-up and investigate how similar this is across the treatment arms to assess possible attrition bias in data collection. The rate of protocol adherence will be reported within the intervention group in terms of participants who adhere to the intervention they were allocated to receive or comply with the scheduled visits.

A baseline table will compare (descriptively) the demographic and key clinical characteristics between the two trial arms. The primary clinical outcome measure at 4 months will be analysed on an intention-to-treat basis (participants analysed according to the arm to which they were randomised, irrespective of whether they actually received the intervention as intended) using a linear regression model adjusted for baseline outcome scores, age and gender to estimate the likely range of intervention effects, i.e. to determine if the mean total CIS-R score differs between the intervention and the usual care arms after controlling for the differences at baseline. The emphasis will be on confidence intervals of effect size estimations, rather than hypothesis testing to explore the imprecision around effect sizes. Mixed-effect ordered logistic regression models adjusted for age and gender will be used to compare the individual symptoms on the CIS-R between the trial arms. Scores on each of the 14 symptom groups on the CIS-R will be entered into the models as ordinal dependent variables. We will also analyse between-group differences in secondary outcomes at 4 months and provide point and 95% interval estimates from linear or logistic regression models as appropriate to the outcome data being analysed (linear for numerical measures and logistic for categorical measures). A descriptive assessment of resource use stratified by treatment arm will also be presented.

### Qualitative data

A process evaluation will be conducted and the data generated will be essential to planning a future definitive RCT. We will assess what the strengths, weaknesses and areas for improvement are in the feasibility study. To assess the acceptability of the intervention to participants, we will conduct up to 20 semi-structured interviews (data will be collected until category saturation is achieved) with participants in the intervention group shortly after the 4-month follow-up. We will also request interviews with those who drop out of the study. These interviews will be conducted by the qualitative researcher. We will sample on baseline characteristics to ensure views from a diverse sample are sought.

Participants will be interviewed to determine their overall perspectives of the intervention with a particular emphasis on how acceptable useful they found the sessions with the support worker. We will ask whether participants attended any groups, and how acceptable and useful they found them. We will also explore barriers and facilitators to their (non-)engagement with the support worker or with groups and determine whether their engagement in a group has continued.

The views of GPs will also be gathered as part of the process evaluation. We will interview up to 12 GPs from the participating practices. GPs will be reimbursed for their time. We will explore GPs’ views and experiences of the intervention and whether it has affected their management of older people with anxiety and/or depression. We will ask the GPs for their views on the roles and contributions of the third sector in supporting this population, including barriers and facilitators to working with that sector.

We will interview the six support workers who participated in the training and delivered the intervention. We will explore their overall views and experiences of working with older people with depression and/or anxiety and how the intervention helped or did not help. We will ask them about their experiences of the training and supervision, and liaison with primary care if this took place. Interviews will be conducted at a venue and time convenient to the participants and are expected to last around 45 min.

Semi-structured interviews will be transcribed verbatim, the transcripts forming the data for analysis. The data will be stored and analysed using the NVivo software. Initially the data will be analysed using the constant comparison method, which is the principal technique in the grounded theory approach [50]. The data will then be presented using the principles of framework analysis [51], as this method is appropriate for applied policy research and the data will allow us to understand how the intervention was implemented by support workers and received by participants. A team of multi-disciplinary researchers will conduct the analysis individually, and then agree themes through discussion.

The results of the free-text participant burden question in the baseline and follow-up questionnaires will also be collated and analysed using a framework approach.

### Fidelity

A sample of the support worker–study participant consultations will be digitally audio recorded. Each support worker trained (*n* = 6) to deliver the intervention will be asked to record a total of two consultations with each participant (ideally the first and second consultations). A digital recorder will be used by the support worker at the start of the consultation. Fully informed consent from the participant for the recording of both consultations with the support worker will be obtained beforehand by a member of the study team. The use of these audio recordings will focus on the fidelity of the intervention delivery, for example which elements of this intervention the support workers used, whether the training was implemented by support workers and whether there are any gaps in intervention delivery.

### Study success criteria

To determine feasibility, findings will be assessed against the following criteria:

- Engage general practices: at least six practices to participate.
- Recruit, train and retain support workers: at least four support workers to be retained.
- High response rates to the screening questionnaire: receive useable responses from at least 40% of those mailed.
- High response rates to the follow-up questionnaire: at least 75% for the primary outcome using minimal data collection.
- Adherence to intervention: data to be drawn from support worker notes and recordings of sessions. Each participant will have at least one contact with a support worker. This will be investigated in more detail with the in-depth qualitative interviews with the participants, support workers and GPs to determine barriers and facilitators to delivering/receiving the intervention.
- Acceptability of the intervention: to be determined from qualitative interview data.

We will also analyse the results of the free-text participant burden question in the baseline and follow-up questionnaires. This will help us to determine the acceptability and feasibility of the questionnaires.

### Monitoring and safety considerations

The NOTEPAD feasibility trial will be monitored in line with the protocol and Keele CTU standard operating procedures. An independent trial steering committee will monitor the progress of the trial and a data monitoring committee will be convened to monitor the safety of the participants and data integrity. Monitoring will also be undertaken by the research ethics committee and the funder in the format of annual progress reports.

A serious adverse event (SAE), as defined by the NHS Health Research Authority, that occurs for a study participant must be reported to the research ethics committee if, in the opinion of the principal investigator, the event resulted from administration of the intervention (it was related to the study) and it was unexpected.

All participants remain under the care of their GP throughout the study. We will ask all participating GPs to report SAEs within 24 h of becoming aware of these. Reporting procedures will be made clear during general practice initiation sessions and will be built into site service level agreements, copies of which will be available within a local site file held at every participating practice. All participants will also be provided with the contact details of the study coordinator and asked to self-report any such events to the NOTEPAD team as soon as possible. Participants randomised to the NOTEPAD intervention will see or speak to the support worker during the intervention. Therefore, we will also ask the support workers to ask about, record and report any potential participant SAEs they become aware of to the team.

A risk protocol has been created for this study should participants express thoughts of suicide or self-harm. Researchers and support workers must initiate the suicide ideation protocol whenever a study participant expresses thoughts of suicide or self-harm. In such cases, the researcher or support worker, with the study participant’s permission, will inform the study participant’s GP and notify the clinical investigator (or nominated deputy).

We will use our standard risk protocol to deal with distress in participants, along with de-briefing, support and supervision of researchers.

### Patient and public involvement and engagement

The study’s design and processes have been informed by PPIE according to INVOLVE’s recommendations (http://www.invo.org.uk/resource-centre/resource-for-researchers/). Participants at our initial meeting endorsed the concept of a non-medicalised approach to the management of depression and anxiety in older people, and welcomed partnering third-sector groups such as Age UK. PPIE members also supported the idea of a one-to-one intervention delivered by a worker from Age UK. We also sought comments on the full application at a further meeting. Members of the group felt that most older people would be happy to talk to the support worker and strongly supported the idea of tailoring activities to the older person’s interests, which is an important part of our strategy. Many felt that some older people might need some gentle encouragement and reassurance to take part. They also felt strongly that barriers to participation, such as transport and lack of confidence, need to be addressed. Both points are at the heart of the intervention.

In subsequent meetings, our PPIE Group provided strong input on our patient information sheets, letters and patient resources. The group approved the NOTEPAD logo and suggested the strapline ‘Supporting Mental Strength’, which we adopted.

## Discussion

The proposed project is a feasibility study determining whether it is possible to train third-sector workers to deliver a psychosocial intervention to depressed and/or anxious older people and whether this is acceptable to patients.

The target population (older people with anxiety and/or depression) means that this study is of strategic importance to the NHS and social care, and given the existing evidence and changes in demography, has the potential to have a significant impact on many people across the UK. By including the public, service users, the voluntary sector and clinicians in its development, the proposed intervention will likely have a high degree of acceptability and validity, and the findings of any definitive trial will have a higher probability of being commissioned than might otherwise be the case.

This study will inform a definitive multi-centre RCT, which has the potential to contribute to an innovative reorganisation of existing resources across health and social care, including the third or voluntary sector, and more effectively target resources to early intervention, to prevent the deterioration of mental health symptoms in older people.

### Dissemination

The results of this study will be reported to the trial steering committee, data monitoring committee and our funder, published in relevant high-quality peer-reviewed journals and presented at both national and international conferences.

## Trial status

Screening for potentially eligible participants commenced in January 2017. Recruitment has completed. The follow-up for this study is due to be completed in August 2017.

## Additional file

Additional file 1: SPIRIT 2013 Checklist. Recommended items to address in a clinical trial protocol and related documents. (DOC 122 kb)

## Abbreviations

BA: Behavioural activation
CASP-12: Control, Autonomy Self-realisation and Pleasure
CIS-R: Clinical Interview Schedule Revised
CTU: clinical trials unit
GAD7: Generalised Anxiety Disorder 7
GP: General practitioner
NHS: National Health Service
NICE: National Institute for Clinical Excellence
NIHR: National Institute for Health Research
PHQ9: Patient Health Questionnaire
PPIE: Patient and Public Involvement and Engagement
RCT: Randomised controlled trial
SAE: Serious adverse event

## Footnotes

1 The Adult Attitude to Loneliness is not yet a validated measure. It was adapted from the Adult Attitude for Grief and it will be used in this feasibility study to gather data on the acceptability of the scale to NOTEPAD participants and to the research nurse who administers it.
